# Clinical significance of HPV-DNA testing for precancerous cervical lesionS

**Published:** 2014

**Authors:** M Moarcăs, IC Georgescu, E Brătilă, M Badea, ECM Cîrstoiu

**Affiliations:** * Obstetrics and Gynaecology Department, University Emergency Hospital, Bucharest; ** Obstetrics and Gynaecology Department, “Sf. Pantelimon” Emergency Hospital; *** Micomi Clinic, Bucharest

**Keywords:** HPV, precancerous cervical lesions, screening

## Abstract

Cervical screening by using cytology was proven efficient in reducing the mortality secondary to cervical cancer, but this method has limitations. High risk HPV infection is essential for cervical cancer development so HPV testing is a new tool used for screening patients for cervical neoplasia. HPV testing was proven most useful for women over 30 years old, in cases in which cytology identified ASC-US and after treatment for CIN. This article outlines the clinical significance of HPV-DNA testing for precancerous cervical lesions and the evidence that stands behind these recommendations.

**Abbreviations:** HPV = Human Papilloma Virus, ASC-US = Atypical Squamous Cells of Undetermined Significance, CIN = Cervical Intraepithelial Neoplasia, LSIL = Low Grade Squamous Intraepithelial Lesion, DNA = Deoxyribonucleic Acid

## Background

The cervical cancer screening by using cervical cytology has significantly reduced the mortality secondary to squamous cell cervical cancer [**1**]. In spite of the results obtained by using cervical cytology, this screening method has multiple limitations that led to the research for the other screening tests. Taking into consideration the fact that the high oncogenic risk HPV infection is essential for the development of precancerous and cancerous cervical lesions, recent studies have focused on the detection of these types of infections [**2**]. As a screening test for cervical cancer, HPV-DNA testing has a high sensitivity but a low specificity compared to cervical cytology [**3**] due to the high prevalence of limited HPV infections in younger women. 

**HPV-DNA testing for women over 30 years old**

Studies have demonstrated that HPV-DNA testing, adjacent to cervical cytology increases CIN3 detection with 7-31% for the first testing, and reduces CIN3 and cancer detection rate for subsequent tests (absolute reduction in cancer rate at second test 0.03-0.05%) [**3**-**5**]. Combined screening is recommended at 5 years interval because the rate of cervical cancers associated with this method is lower than the one associated with the 3 yearly cervical cytology [**6**]. The 5 years cervical cancer incidence is 3,2 per 100000 women per year for women who have negative HPV and cytology, 3,8 per 100000 women per year for women with negative high risk HPV test only, and 7,5 per 100000 women per year for women with negative cytology only [**6**]. Abnormal cytology for women with positive high risk HPV test increases the 5 year risk of CIN3 and cervical cancer from 5,9% to 12,1% compared to women with a normal cytology and a positive HPV test. 3 yearly combined testing is associated with a risk for cervical cancer similar to 5 yearly combined testing (0,39% compared to 0,61%) but is associated with a significantly higher number of negative colposcopies [**7**]. 

A combined screening technique, by using HPV-DNA testing and cervical cytology, increases the detection rates for cervical adenocarcinoma, a very important benefit taking into consideration that unlike squamous cell cervical carcinoma, the invasive cervical adenocarcinoma incidence has not reduced substantially after the initiation of cervical screening by using cytology [**6**,**8**]. A positive high risk HPV test is highly associated with cervical adenocarcinoma (OR-Odds Ratio 81,3) [**9**]. 

**HPV-DNA testing in patients with ASC-US**

The cytological interpretation of ASC-US represents a category of morphologic uncertainty. The definition of ASC-US is “some, but not all” the features of a LSIL and as such, includes both poorly sampled and poorly represented LSIL and the many morphologic mimics of LSIL [**10**]. For patients with this result, other tests are necessary in order to determine the risk for precancerous cervical lesions. For women with positive high-risk HPV test and ASC-US cytology, colposcopy is recommended because these women have a 2 years risk of CIN3 and invasive cervical cancer of 10% [**11**]. Studies have demonstrated that for women with ASC-US and negative high-risk HPV test, the risk for precancerous cervical lesions is very low, similar to women with negative cytology and HPV test. The absolute risk of CIN3 for women with ASC-US and negative HPV is 0,28% at that moment and 0,54% at 5 years [**6**,**12**]. Because this risk is less than 1% for these women, it is recommended to return to routine cervical screening [**10**]. 

**HPV-DNA testing after CIN treatment**

CIN treatment reduces the risk of invasive cervical cancer with over 90%, but these women still have a 5 times higher risk of developing invasive cervical lesions compared to women with normal cytology because treatment does not always manage to eliminate the HPV infection [**13**]. These patients need postop follow up in order to reduce the risk of invasive lesions. Old follow up guidelines recommended yearly cytology after CIN treatment. This type of follow up is excessive for approximately 90% of the patients and patients compliance over such a long period is low [**14**]. Also cervical changes after CIN treatment can make cytology evaluation difficult especially in case of cervical stenosis. High risk HPV testing for these patients increases the efficiency of the screening programme.

A British study has demonstrated that only 0,2% of women with negative HPV and cytology post treatment had CIN2 at 5 years and only 2,4% of women with negative HPV test and inadequate cytology or ASC-US post treatment had CIN2 at 5 years. In conclusion, 97,6% of the women with ASC-US and negative high risk HPV have not developed CIN2 at five years, so colposcopy can be deferred for these patients [**15**]. 

Kocken et al. evaluated the efficiency of cytology and HPV testing follow up for women with cone biopsy for CIN2. 6 months post treatment high risk HPV testing had a higher sensibility than cytology testing (relative sensibility 1,15, CI 95% 1,06-1,25) without a reduction in specificity (relative specificity 0,95, CI 95% 0,88-1,02) [16]. Combined high-risk HPV testing and cervical cytology had a higher sensitivity for the detection of recurrent high-grade cervical lesions than cytology or HPV testing alone. In order to increase the screening programme sensitivity it is recommended to repeat the testing at 18-24 months [**14**]. 

Genotype specific testing is important because the 6 months post cone biopsy persistence of the same type HPV is an independent risk factor for CIN recurrence at 24 months. Unlike the general high risk HPV testing, the type of specific testing predicts residual disease with similar sensitivity but higher specificity (relative specificity 1,43%) [**17**]. The persistence of HPV 16 post treatment should lead to a more intense monitoring because this HPV type has lower disappearance rate compared with the other high risk HPV types [**18**]. 

## Conclusions

The use of high risk HPV testing as a screening tool increases the detection rates for all the precancerous and cancerous cervical lesions and decreases the screening interval in a safe manner. Also, HPV testing for women with ASC-US cytology reduces the number of unnecessary colposcopies by underlying the women who are at high risk of cervical precancerous lesions. Post CIN treatment, high-risk HPV testing points out the women who are at risk of recurrence, reducing the number of women who require frequent and long-term follow up. 

## References

[1] Parkin DM, Bray F (2006). Chapter 2: The burden of HPV related cancers. Vaccine.

[2] de Sanjose S, Quint WG, Alemany L (2010). Human papillomavirus genotype attribution in invasive cervical cancer: a retrospective cross-sectional worldwide study. Lancet Oncol.

[3] Naucler P, Ryd W, Tornberg S (2007). Human papillomavirus and Papanicolaou tests to screen for cervical cancer. N Engl J Med.

[4] Bulkmans NW, Berkhof J, Rozendaal L (2007). Human papillomavirus DNA testing for the detection of cervical intraepithelial neoplasia grade 3 and cancer: 5-year follow-up of a randomised controlled implementation trial. Lancet.

[5] Ronco G, Giorgi-Rossi P, Carozzi F (2010). Efficacy of human papillomavirus testing for the detection of invasive cervical cancers and cervical intraepithelial neoplasia: a randomised controlled trial. Lancet Oncol.

[6] Katki HA, Kinney WK, Fetterman B (2011). Cervical cancer risk for women undergoing concurrent testing for human papillomavirus and cervical cytology: a population-based study in routine clinical practice. Lancet Oncol.

[7] Stout NK, Goldhaber-Fiebert JD, Ortendahl JD, Goldie SJ (2008). Trade-offs in cervical cancer prevention: balancing benefits and risks. Arch Intern Med.

[8] Bray F, Carstensen B, Moller H (2005). Incidence trends of adenocarcinoma of the cervix in 13 European countries. Cancer Epidemiol Biomarkers Prev.

[9] Castellsague X, Diaz M, de Sanjose S (2006). Worldwide human papillomavirus aetiology of cervical adenocarcinoma and its cofactors: implications for screening and prevention. J Natl Cancer Inst.

[10] (2012). ACS-ASCCP-ASCP Screening Guidelines. American Society for Colposcopy and Cervical Pathology Journal of Lower Genital Tract Disease.

[11] ASCUS-LSIL Triage Study (ALTS) Group (2003). A randomized trial on the management of low-grade squamous intraepithelial lesion cytology interpretations. Am J Obstet Gynecol.

[12] Stoler MH, Wright TC Jr, Sharma A, Apple R, Gutekunst K, Wright TL (2011). High-risk human papillomavirus testing in women with ASC-US cytology: results from the ATHENA HPV study. Am J Clin Pathol.

[13] Soutter WP, De Barros LA, Fletcher A, Monaghan JM, Duncan ID, Paraskevaidis E (1997). Invasive cervical cancer after conservative therapy for cervical intraepithelial neoplasia. Lancet.

[14] HPV testing in the follow-up of women post colposcopy treatment, Cervical Check. The National Cervical Screening Programme, Ireland.

[15] Mesher D, Szarewski A, Cadman L, Cubie H, Kitchener H, Luesley D (2010). Long-term follow-up of cervical disease in women screened by cytology and HPV testing: results from the HART study. Br J Cancer.

[16] Kocken M, Uijterwaal MH, de Vries AL (2012). High risk human papillomavirus testing versus cytology in predicting post-treatment disease in women treated for high-grade cervical disease: a systematic review and meta-analysis. Gynecologic Oncology.

[17] McCredie MR, Sharples KJ, Paul C (2008). Natural history of cervical neoplasia and risk of invasive cancer in women with cervical intraepithelial neoplasia: a retrospective cohort study. The Lancet Oncology.

[18] Heymans J, Benoy IH, Poppe W, Depuydt CE (2011). Type specific HPV geno-typing improves detection of recurrent high-grade cervical neoplasia after conisation. International Journal of Cancer.

